# Cultural beliefs of time orientation and social self-construal: Influences on climate change adaptation

**DOI:** 10.4102/jamba.v10i1.510

**Published:** 2018-10-25

**Authors:** Aida C. Terblanche-Greeff, Jon-Vegard Dokken, Dewald van Niekerk, Ruth A. Loubser

**Affiliations:** 1 Unit for Environmental Sciences and Management, North-West University, South Africa; 2 Department of Sociology and Human Geography, University of Oslo, Norway; 3 School of Philosophy, North-West University, South Africa

## Abstract

Climate change is one of the greatest challenges humankind faces and adaptive behaviour is an imperative response to such change. Culture and the resulting worldview are determinants of behaviour and eminent cultural beliefs are that of time orientation (TO) and social self-construal (SSC). To date, no research focuses on these beliefs from an indigenous South African perspective or the manner in which it may subsequently affect a community’s adaptation towards climate change. Q-methodology was used to study perspectives and beliefs in three peri-urban communities in South Africa and to investigate the interrelation between themes such as TO, SSC, climate change awareness and climate change causality. It became apparent that the communities are aware of climate change, yet little to no efforts are currently being made to adapt to climatic change. This absence of motivation to adapt may be attributed to limited risk perception and cultural beliefs of TO and SSC. This study aims to contribute to the understanding of cultural beliefs and its impact on climate change adaptation behaviour in the South African context. It is concluded that TO and SSC from an indigenous South African perspective influence community adaptation to climate change.

## Introduction

In the last two decades, disasters that are worsened by climate change and induced by anthropogenic or natural hazards continue to ‘claim millions of lives, affect billions of people, and cause trillions in economic losses’ (Van Niekerk & Terblanché-Greeff 2017:3). As such, climate change and its dire consequences are a reality that all of humanity will have to face.

Humans are in essence culturally orientated and a society’s culture is embedded in all social spheres. For this reason, the actions of a community’s members will be determined by its collective cultural beliefs, and such sociocultural traits will greatly influence adaptive behaviour regarding climate change (Adger et al. 2009; Wolf 2011). Cultural beliefs (e.g. time orientation [TO] and social self-construal [SSC]) will affect the manner in which community members identify risks caused by climate change (McNeeley & Lazrus 2014). Risk identification is important as a level of risk must be perceived to encourage cognition of possible adaptive behaviour and effects (Schwarzer 1992). This is specifically true for disaster risk reduction (DRR) efforts as the community’s cultural beliefs will determine whether risks warrant the required adaptive behaviour. Consequently, the lack of justification for actions caused by the level of risk perceived can impede the community’s ability to adapt to climate change (Adger et al. 2009).

Climate change is defined as (IPCC 2012):

a change in the state of the climate that can be identified (e.g. by using statistical tests) by changes in the mean and/or the variability of its properties and that persists for an extended period, typically decades or longer. (p. 557)

The negative effects of climate change are an inescapable reality and the potentially disastrous impact on Africa could be immense because of the continent’s high levels of vulnerability (Beg et al. 2002).

Neil Adger (2006:268) attempts to clarify *vulnerability* as ‘the state of susceptibility to harm from exposure to stresses associated with environmental and social change and from the absence of capacity to adapt’. Nonetheless, defining the concept *vulnerability* is complex as climatic changes, risk, hazards and coping capacities are region-specific, and in various regions and local communities, several groups may have an inclination to be more vulnerable to climatic change and less capable to adapt thereto (O’Brien, Sygna & Haugen 2003). Therefore, it will be useful to contextually identify vulnerable communities to determine appropriate adaptation capacities, especially when considering that a community’s level of vulnerability and adaptation capacity relate to political, economic and sociocultural milieu (UNICEF 2011).

The research presented was conducted as part of the South Africa–Norway Research Co-operation (SANCOOP) project which focused on subjective beliefs and climate change adaptation. Currently, no research has been conducted where focus is allocated to SSC and TO based on a specific South African perspective. The aim of the research was to investigate the cultural beliefs (specifically TO, and SSC) of three peri-urban communities in the North-West Province of South Africa to determine its impact on climate change adaptation.

To surmise, this article focuses on the cultural beliefs of TO and SSC, investigates how such beliefs present in the indigenous South African perspective and considers how the context-specific beliefs may affect the community’s climate change adaptation. A brief conceptual framework familiarises the reader with themes such as TO, SSC, risk perception and climate change adaptation. Secondly, the empirical research section focuses on methodology, sampling, data collection and data analysis. From there, results offer abductory findings based on the qualitative analysis of quantitative data. Lastly, a conclusive discussion reveals how the indigenous South African perspective (specifically the cultural beliefs of TO and SSC) influences the community’s adaptation to climate change, and this section offers suggestions to facilitate DRR.

### Conceptual framework

The aim of this framework is to lay the conceptual foundation for the qualitative analysis of the statistical data as per Q-methodology, and this will facilitate the study of cultural beliefs from an indigenous South African perspective and how these beliefs affect a community’s climate change adaptation. As such, the framework introduces the concepts: worldview and culture; TO (more specifically polychronism, and Mbiti’s African TO); and SSC (more specifically the cultural value of *Ubuntu*). Thereafter, a neologism, *Afro-polychronism*, presents and elucidates cultural beliefs of TO and SSC as found in the indigenous South African perspective. The last section of the conceptual framework discusses links between cultural beliefs, risk perception, climate change adaptation and DRR.

### Worldview and culture

The concepts of worldview and culture are interwoven, which becomes evident when these concepts are explained. A worldview is a subjective perception and systematic organisation of reality. Members of a sociocultural group agree on this reality and, subsequently, their beliefs, perspectives and attitudes originate from the established communal worldview (Kraft 1979). Similarly, the concept of culture is described as ‘systems of belief and practices that are built upon the implicit assumptions that people make about themselves, about the world around them and about ultimate realities’ (Hiebert 1998:171). As such, the concepts of worldview and culture pertain to how we make sense of the world, which will ultimately influence our being-in-the-world and being-with-others. As the meaning and description of the concepts worldview and culture are similar, the terms will be used interchangeably in the article.

Culture is transferred from one generation to the next, and culture (as systems of beliefs) demarcates the prescribed behaviour of members of a sociocultural group (Douglas 1978; Olteda et al. 2004). More specifically, the manners in which people act are guided by the interaction between community members, and this interaction is influenced by various cultural beliefs (Legohérel et al. 2009). The cultural beliefs – convictions or tenets that people consider to hold true – that were investigated in this study are TO and SSC.

### Time orientation and polychronism

A common cultural belief embedded in a community’s worldview is the notion of time. Social practices formulate temporality by ascribing meaningful codes to it which in turn makes it easier to organise events in time. Zimbardo and Boyd (1999) concur and define time perspective as:

the often nonconscious [*sic*] process whereby the continual flows of personal and social experiences are assigned to temporal categories, or time frames, that help to give order, coherence and meaning to those events. (p. 1271)

The concept of time orientation (TO) refers to the subjective perspective allocated to temporality. TO denotes the perspective of time in which structure is allocated to temporality based on the subjective attitude of an individual or group (Zimbardo & Boyd 1999). Cultural TO influences the focus on various time structures, whether it is past, present, future or a combination of these views (Mello, Finan & Worrell 2013). A prominent trait of TO is its inseparability from culture as TO is formed by the individual’s or group’s worldviews (Fulmer, Crosby & Gelfand 2014; Offe 2001). The meaning allocated to time frames and the relevance of the past, present and future will vary widely as a result of factors such as life experiences, social class, education, religion, familial relations, et cetera. (Güell & Yopo 2016; Offe 2001).

Anthropologist, Edward Hall (1959, 1976, 1981, 1990), is considered by scholars to be one of the first researchers to study communities based on the manner in which group members utilise time. He categorised communities as either monochronic or polychronic, and these time perspectives became the dominant categories when studying the TO of various cultural communities.

In monochronism, time is experienced in a linear manner and can be explained as a line extending from the past timeframe in the direction of the future timeframe (Hall & Hall 1987). This line extension is made up of the parts time is segmented into, for example, seconds, minutes, hours, days, weeks, etc. (Booth 1975). This segmentation facilitates the compartmentalisation and scheduling of time, making it possible for the individual to focus on activities in succession (Hall & Hall 1987). In monochronism, every instance of time can be experienced but once; and time is considered empty and it must be filled with occurrences (Offe 2001). Therefore, it is the individual’s responsibility to utilise time wisely and this can be done by controlling and scheduling time.

Monochronism also provides a personal identification category as it influences the individual’s sense of self. In linear monochronic time, the autonomous individual must feel that he or she has progressive continuousness – the individual must reason in the present time frame that he or she will, in fact, exist in the future (Kirsch 1988). Lowenthal (1992) concurs by asserting that ‘in planning ahead we try to make something of ourselves’. Here the focus on future events is formed by meaningful conceptual representations of such events, and these representations and anticipations will motivate behaviour (Milfont, Wilson & Diniz 2012).

In a dichotomous manner, polychronism focuses more on the qualitative nature of time. It is a TO whereby multiple tasks and proceedings are conducted simultaneously in the same time block and these actions are characterised by a high level of community participation where the focus is placed on time-consuming, yet meaningful, relations between individuals (Hall & Hall 1987). The emphasis allocated to quality human relationships and interaction is ascribed to the undeniable collectivistic trait of polychronism.

Various population groups (Mediterranean, Latin American, Far Eastern and African) are categorised as polychronic (Hall & Hall 1987) regardless of the fact that these communities each have unique cultural characteristics. Polychronism in Africa is often termed *African time* and this temporal concept refers to the cultural behaviour of Africans where activities are not rushed and limited adherence is allocated to deadlines because of the focus on communality (Dissel 2007).

### Mbiti’s *African time* orientation

John Mbiti is the first African scholar to bring the concept of *African time* into the academic arena, which he did through his seminal work *African Religions and Philosophy* (1969). For Mbiti (1969) the dominant African TO has distinctive characteristics that exemplify its contrast to contemporary monochronic TO. Time from the African perspective can be generated by mankind and therefore it is an instrument that can be exploited. Humans are not imperilled or governed by time and temporality cannot be conceived as a commodity. Instead of commodifying time and labelling it in terms of numerical occurrences, the value and significance of time are centred around communal experiences. *African time* is concrete and cyclical, therefore time renews daily. Time is boundless and based on the *African time* perspective time cannot ‘run out’.

Mbiti (1969) goes further and elucidates African TO through the concepts *actual time, potential time* and *no-time*. Based on the cyclical nature of African time, temporality is regarded as the summation of experienced events. The sum of events represents past and present instances, with the flow of time being in a backward direction from present to past. This forms the concept of *actual time* as defined by Mbiti (1969). Ikechukwu Kanu (2015:130) links the importance of the past and present in African TO with the Heideggerian notion of *Vergangenheit Vergesenheit* (forgetfulness of the past), and therefore, if the present suffers from the amnesia of the past, ‘there would be the death of sein (being), for its life is inseparable from its past and the forgetfulness of the past leads to the decay of the present’. As such, Mbiti’s (1969) African TO allocates limited importance to future events because of the primary focus on *actual time* as constituted by past and present events. Nonetheless, even though limited attention is attributed to future events, it is not dismissed in totality.

Future events are divided and described by two terms: *potential time* and *no-time*. Unavoidable predictable occurrences that ‘fall in the rhythm of natural phenomena’ delineate *potential time* (Mbiti 1969:16). Examples of such phenomena are seasonal cycles and climatic changes. In contrast, unknown and indistinct future occurrences that are yet to be experienced cannot be representative of time in the African temporal orientation, and this idea of the future is described as *no-time*.

At first glance, Mbiti’s African TO appears to be three-dimensional (past, present and future), when, in fact, it is two-dimensional (past and present). This is because of the abstraction that future events (*potential time* and *no-time*) are unable to constitute time as it has not been experienced yet. This limited future view is near-sighted, as ‘future’ can only anticipate (to some degree) by events no more than 2 years into the future.

Scott Moreau (1986) uses the following analogy to better illustrate Mbiti’s concept of African TO. An individual is standing in a river, facing the downstream flow of the water. The water current represents the flow of time (*actual time*) and the individual’s temporal view is represented by his or her peripheral perspective, hereby including all the water that has moved downstream past him or her. The individual’s ‘future’ view is represented by what can be visually perceived upstream (near-sighted future view) and unseen water (representing *no-time*) is insignificant for the individual because the unseen water (unknown future events – *no-time*) will ‘pass when and how it passes, and then it will become of consequence’ (Moreau 1986:39). Only the water (time flow) in peripheral view that is currently flowing past the individual and the water that has previously passed the individual (past and present timeframes constituting *actual time*) carry significance. Natural debris (like sticks and leaves) floating in the water is representative of inevitable natural phenomena (*potential time*), and the debris will unavoidably float past him or her. The individual has no logical motivation to move upstream and therefore remains stationary. Awareness is attributed to the fact that the upstream current will eventually reach him or her and moving against the current will not accelerate the waters’ natural flow. Therefore, it is considered unnecessary to focus on the uncontrollable flow of the upstream water (unknown future events: *no-time*). Moreau (1986) concludes this analogy by stating that an individual with a contemporary monochronic TO will see no use in remaining stationary, but will rather swim upstream, thus chasing the future.

In addition to the aforementioned characteristics, African TO is denoted by time-consuming interactions between individuals that lead to the creation of meaningful social relations (Kudadjie 1996). As such, African TO is decidedly polychronic. Cognisance of Mbiti’s African TO is essential as it informs social identity and represents a sociocultural reality (Babalola & Alokan 2013).

### Social self-construal – *Ubuntu* collectivism

Beliefs surrounding TO provides the structural foundation for cultural character, including the individual’s self-construal, which refers to the perception and interpretation of the individual self in social contexts (May 2008). The awareness of the self in social contexts is described by self-construal that ‘regulates personal experiences in social interactions and interpersonal relations’ (Han, Kim & Yoshiyuki 2016:38). Importantly, when investigating the influences cultural beliefs will have on behaviour patterns, cognisance must be allocated to the community’s SSC in concurrence with TO as both influence attitudes towards being-with-others (Güell & Yopo 2016).

A prominent SSC categorisation in South Africa is collectivism as presented by the cultural concept of *Ubuntu*, which is often described by the maxim ‘*A person is a person through other persons*’. Here, an individual is a ‘being-in-relation’ and a sense of belonging is based on relationality (Otÿele 1991:9). Concurrently, the African proverb ‘Go the way that many people go; if you go alone, you will have reason to lament’ illustrates that an individual’s cultural security lies in the personal association and contributive role played within a group (Matondo 2012:39). This interdependency facilitates the sharing of responsibilities and roles between members, thus enabling growth and progress of the group (Jackson 2010).

An individual’s SSC and personhood can and should be developed through moral interactions with others. Supportively, Mvuselelo Ngcoya (2009) states that:

Ubuntu stresses the importance of community, solidarity, caring and sharing. This worldview advocates a profound sense of interdependence and emphasizes that our true human potential can only be realized in partnership with others. (p. 1)

Therefore, communal interchange and compassion are integral in *Ubuntu*. The ultimate life goal should be to become a person, that is, ‘a (complete) person, a (true) self or a (genuine) human being’ where ‘one can be more or less of a person, self or human being, where the more one is, the better’ (Metz 2011:537).

It is apparent that an individual cannot attain *Ubuntu* in isolation, but rather through interdependency and meaningful interactions with others. *Ubuntu* collectivism is characterised by ‘identity’ and ‘solidarity’. Community members will identify as ‘I’ in ‘We’ which serves as behavioural motivation to reach communal goals; and goal achievement is attained through solidarity by means of mutual aid, altruism and sympathy (Metz 2011).

The highly holistic and collective nature of *Ubuntu* is indicative of polychronic temporal orientations where time is utilised to create relations between group members, and time is used to promote group progress and growth.

From the preceding thematic discussions, it is apparent that TO (specifically polychronism and Mbiti’s African TO) and SSC (relational rationality of *Ubuntu*) are reciprocal cultural beliefs. The unique temporal orientation (*African time*) and the context-specific polychronism (*Ubuntu*) prominent in the African perspective are interlinked to a degree of inseparability and therefore a synthesised concept, *Afro-polychronism*, is formulated. Additional motivation for the concept formulation stems from the fact that collectivistic polychronism, as found in the African populace, cannot be applied to any other polychronic groups owing to the unique context-specific cultural traits of *Ubuntu* as rooted in the African perspective. Other polychronic communities may share comparable temporal perspectives with the African perspective, but they may also have cultural qualities that differ from the cultural aspect of *Ubuntu*.

### *Afro-polychronism* in the indigenous South African culture

For the purpose of this study, it is necessary to categorise the investigated indigenous South African perspective based on the cultural beliefs of TO and SSC, as this will influence behavioural patterns. A more context-specific neologism – *Afro-polychronism* – is introduced to distinguish this worldview and its unique traits from other population groups that may have similar but not truly identical cultural beliefs. As motivation for the neologism, it is argued that some aspects of polychronism, Mbiti’s African TO and *Ubuntu* collectivism as cultural beliefs are inseparable and reciprocal, that is, the way time is perceived and used will influence social interaction and self-awareness, and *vice versa*.

*Afro-polychronism* is a TO which focuses on the past and present as a unified timeframe, and limited value is attributed to the future. Here, the future is not entirely ignored; however, events that fall outside of the natural rhythm of nature are only considered and anticipated in the near-sighted future. The past and present are not successive timeframes but rather coexist (like Mbiti’s *actual time*), and forgetfulness of the past will greatly impact the individual’s present.

Shared experiences in this unified timeframe create a sense of belonging, which is prevalent in collectivistic *Ubuntu*. Thus, past experiences provide wisdom in the present timeframe as these experiences contribute to collectivistically orientated SSC. Collectivism, as found in *Ubuntu*, focuses on group participation and the development of meaningful relationality between people. From this, it is deduced that the adherence to schedules and ‘clock time’ are of less importance when compared to quality interactions between people (characteristic of polychronism). Attention to relationality increases social capital, creates a sense of belonging and forms a specific collectivistic SSC.

TO and SSC as embedded cultural beliefs found in *Afro-polychronism* create a system of sense-making in reality and the investigation thereof has the potential to clarify behavioural patterns of the group holding this worldview. Cultural beliefs not only influence all human behaviour but also human cognition and perceptions. Therefore, TO and SSC as encapsulated by *Afro-polychronism*, will affect the community’s structure of reality, and subsequently the risk that will be perceived regarding climate change.

### Perceived risk and climate change adaptation

In the context of DRR and climate change adaptation, the study of cultural beliefs is crucial based on the increased need for glocal action (Lorenzoni & Hulme 2009). The relation between DRR, culture and risk perception are clear when Aaron Wildavsky and Karl Dake (1990:42) claim that culture can ‘predict and explain what kind of people will perceive which potential hazards to be how dangerous’. With that being so, cultural beliefs will influence the community’s level of concern regarding climate change (Adger et al. 2013; McNeeley & Lazrus 2014). Hence, as cultures differ, it is useful to focus on unique and unfamiliar beliefs when identifying strategies of DRR and climate change adaptation.

In DRR practices, behavioural changes must be implemented when addressing community vulnerability regarding climate change and this will be dependent on the perceived risk. Risk perception refers to subjective judgement about the felt probability of experiencing hazards when objective information is lacking or minimal (Gierlach, Belscher & Beutler 2010). Supportively, Dewald Van Niekerk and Aïda Terblanché-Greeff (2017:4) assert that ‘the assessment of various risks associated with natural and anthropogenic hazards, the likelihood of their occurrence, and the conditions of vulnerability, which could be exploited by such hazardous impacts’ are elemental in DRR. When the likelihood of being influenced by a risk is perceived as limited, the motivation for adaptive behaviour will be absent. Accordingly, some level of risk pertaining to climate change must be perceived to facilitate cognisance of the positive effects of adaptive behaviour. The awareness of the favourable consequences will subsequently motivate the needed behavioural modifications for climate change adaptation (Grothman & Patt 2005).

Cultural beliefs (e.g. TO and SSC) will determine the level of priority allocated to perceived risk and adaptation, which will affect the individual’s social involvement (Van Niekerk & Terblanche-Greeff 2017). Subjective judgement, choices and behaviour will be affected by TO (Dissel 2007; Zimbardo & Boyd 1999). Encapsulated in the concept of time are the beliefs regarding future occurrences. Because future considerations differ across cultures, it will be valuable to investigate a community’s future view (McInerney 2004; Wallman 1992). This is imperative as climate change adaptation requires present action to influence future consequences.

## Empirical research

Q-methodology was implemented for the empirical investigation of the study. William Stephenson (1953) developed Q-methodology to enable the investigation of subjectivity in an objective manner (Ramlo & Newman 2011). It is a research method that amalgamates qualitative and quantitative techniques as it ‘encompasses a distinctive set of psychometric and operational principles … conjoined with statistical applications of correlational and factor analytic techniques, (thus providing) researchers with a systematic and rigorously quantitative procedure for examining the subjective components of human behaviour’ (McKeown & Thomas 2013:ix). Ultimately, the research method has a quantitative procedure to extract qualitative subjectivity and opinions as communicated through self-reference by the research respondents (Cross 2004; McKeown & Thomas 2013). Here subjectivity refers to behavioural patterns that form an individual’s perceptions, beliefs, attitude, opinions or point of view in a current situation, for example, perceptions of risk (Stephenson 1953).

As Q-methodology illuminates the diversity and complexity of subjectivity in an objective manner, it can facilitate thematic identification and analysis of the research topic (Brown 2004; Shinebourne & Adams 2007). It also enables the identification of ‘particular combinations or configurations of themes’ (Watts & Stenner 2005:70) as well as ‘commonalities and differences in subjective perceptions across a sample group’ (Brown 2004:1).

Q-methodological research involves various steps: concourse identification, Q-sample development, Q-sorting, and interpretation and analysis (van Excel & de Graaf 2005). These procedures are supported by the interrelated elements of Q-methodology such as ‘technique (Q-sorting), analytic methods (correlation, factor analysis, and computing factor scores), and methodology (a comprehensive logic of inquiry drawing on behaviourism, indeterminacy, quantum theory, and abductory logic)’ (McKeown & Thomas 2013:88).

It is against this backdrop that Q-methodology was identified as an adequate research design when investigating themes such as *Afro-polychronism* (more specifically TO and SSC), climate change awareness and climate change causality. This research method is also useful in the investigation of cultural beliefs and its influences on risk perception and consequently climate change adaptation, as investigating subjectivity and its components can provide insight into human behaviour (Paige 2014; van Excel & de Graaf 2005).

### Sampling

In Q-methodology, participant selection can be based on theoretical or pragmatic considerations (McKeown & Thomas 2013). Thus, individuals are selected based on their relevance to the study’s subjective dimensions and goals; or based on availability. As Q-methodology aims to explicate the beliefs and attitudes held by an individual or a specific group of participants, there is no need for large sample sizes (Watts & Stenner 2005). Subsequently, no effort is needed to ensure statistical representativeness based on respondent traits like age, gender, religion, race, etc. (Du Plessis 2005; McKeown & Thomas 2013).

For this study, samples from the identified target population were randomly selected from three peri-urban communities (Ikageng, Jouberton and Ventersdorp in the North-West Province of South Africa). These communities were identified based on their peri-urban status and respondents (aged 18–65) were randomly identified by community gatekeepers based on availability. In South African peri-urban areas, low economic status is prevalent and these communities’ exhibit higher levels of vulnerability when faced with climate change. Additionally, the peri-urban communities were used based on the assumption that they might exhibit more indigenous African cultural beliefs as compared to contemporary culture exhibited in urban communities. This indigenous culture is elemental when investigating *Afro-polychronism* and its influence on adaptation to climate change.

The research was divided into three phases. During Phase 1, semi-structured interviews were conducted with 103 respondents from the three communities. For the purpose of Phase 2, a P-sample of 51 respondents were randomly selected from the original sample based on availability.^1^ The P-sample for Phase 3 was selected in the same manner and consisted of 25 respondents.

### Data collection

For Phase 1 of the research, semi-structured interviews were conducted with the respondents at the different peri-urban locations. During these interviews, two open-ended questions were posed: ‘What do you think about the climate?’ and ‘Do you think it will be possible to change your beliefs about the climate?’ Responses were recorded, transcribed and translated into English and formed the concourse used in Q-methodology. To elaborate, the concourse is the process whereby possible statements made by respondents regarding specific themes (e.g. climate, and beliefs) are collected which in turn represent discourse aspects (van Excel & de Graaf 2005). From the concourse, a set of 40 representative Q-sort statements (or Q-sample) were identified and selected, which was presented to the respondents in Phases 2 and 3.^2^

During both phases, the respondents were instructed to sort the Q-sample by means of Likert scaling based on the respondent’s attitude regarding the thematic statements, a process known as Q-sorting. The Likert-scale ranges are Strongly Agree (+3), Agree (+2), Slightly Agree (+1), Neutral (0), Slightly Disagree (−1), Disagree (−2) and Strongly Disagree (−3).

Noteworthy, the conditions of instruction were different in both phases. For the free distribution used in Phase 2, no prescribed distribution diagram was used and the respondents had the freedom to sort the Q-sample without limitations to each scale range. During Phase 3, the respondents were instructed to use the forced-distribution method as illustrated by the diagram represented by Figure 1. Respondents were requested to limit the allocation of statements (according to the diagram) to the prescribed amount *per* Likert-scale range.

**FIGURE 1:**
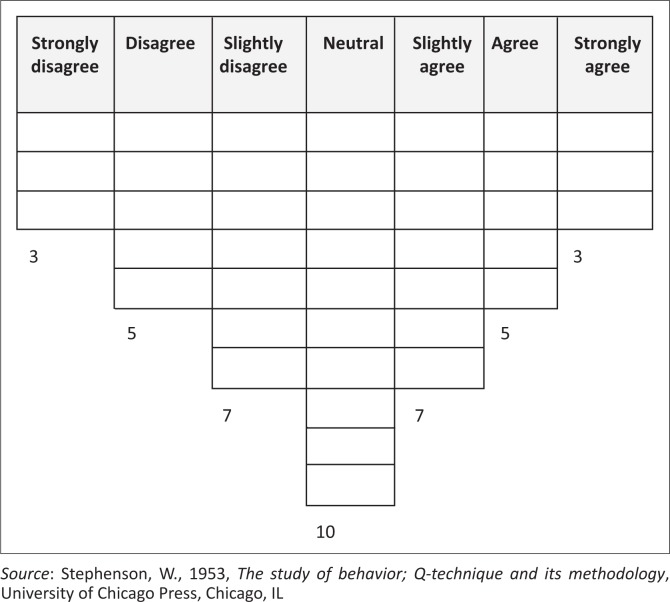
Forced-distribution Q-sorting diagram.

After the Q-sorting sessions (in both phases), the placements of the Q-sample on the Q-sorting diagram were recorded for factor analysis in order ‘to reveal the way groupings of people think similarly or divergently’ (Paige 2014:639).

### Data analysis

The *PQMethod* software programme (version 2.35) was used for data analysis and provided the statistical results and factor arrays on which interpretations and conclusions are based. During the Q-sort analysis of the data collected for the project, five factors (see Appendix 2 and Table 1) were produced that representatively demonstrate attitudes regarding climate and cultural beliefs. These five factors account for 58% of the variation in the Q-sort sample. Factor 1 contributes 20% to the variance, Factor 2 contributes 12%, Factors 3 and 4 contribute 10% each and Factor 5 contributes 6%.

**TABLE 1:** Narrative description of factor arrays 1 and 4.

Factor (Worldview)	Description and/or factor narrative
Factor 1: Collectivist and/or liberal	‘The climate change we experience today is not a punishment for people’s sins and neither is it a sign that the world is ending, but rather a natural occurrence where nature wants to reshape itself. Since the climate affects people’s emotions, it can also cause people to change their beliefs. If we unite we have better chance at solving climate problems and influencing the next generation’s attitudes towards nature. This is important since the climate influences the growth of crops and production of food, and we have to act now to prevent further changes to the climate’.
Factor 4: Technology and/or human	‘The climate plays an important part in our lives and we need to respect the environment. We have the right to know about climate issues that affect us directly and indirectly. Even though the climate is changing, it’s not caused by population growth and is not a sign that the world is ending. Sometimes I think that traditional healers can cause the climate to change, but I also believe that the changes in the climate are related to the burning of fossil fuels and people damaging the environment when they are trying to make money. This means that we should rather try to use sustainable technologies, since this would benefit the environment. It may not be possible for humans to control the climate through technology, but if we work together, we can make a difference’.

Note: For further contextualisation of Table 1, refer to Appendix 2.

## Results and discussion

Of the 40 Q-sort statements selected from the concourse, 12 statements (see Appendix 3 and Table 2 to 5) were identified to enable the investigation of *Afro-polychronism* (TO and SSC) and the possible impact thereof on climate change adaptation. For the purpose of this article, the relevant results, as obtained from the forced-distribution Q-sort in Phase 3, are presented. Abductions are based on the relevant factor arrays of Q-sort statements; the correlation between the factor arrays of various Q-sort statements; the analysis of the five factors (worldviews) and the narrative description thereof; and qualitative accounts provided by the respondents.

**TABLE 2:** Factor arrays of Q-sort statements related to Afro-polychronic collectivism.

No.	Q-sort statement	Factor array 1	Factor array 4
27	We can solve climate problems when we stand together and unite.	Strongly agree	Strongly agree
35	In order to change our beliefs about the climate, we must sit down and discuss the matter.	Agree	Slightly agree
22	Young people can help older people catch up with new knowledge about the climate.	Neutral	Agree

Note: For further contextualisation of Table 2, refer to Appendix 3.

**TABLE 3:** Factor arrays of Q-sort statements related to Afro-polychronic time orientation.

No.	Q-sort statement	Factor array 1	Factor array 4
18	The climate was not better when I was younger.	Disagree	Slightly disagree
19	We can solve environmental problems by returning to the ways of the past.	Slightly disagree	Slightly agree
1	The climate is a natural part of the world we just have to accept and live with.	Neutral	Slightly disagree

Note: For further contextualisation of Table 3, refer to Appendix 3.

**TABLE 4:** Factor arrays of Q-sort statements related to climate change awareness.

No.	Q-sort statement	Factor array 1	Factor array 4
4	The climate is not changing.	Slightly disagree	Strongly disagree
5	There’s something wrong with the climate.	Neutral	Agree

Note: For further contextualisation of Table 4, refer to Appendix 3.

**TABLE 5:** Factor arrays of Q-sort statements related to climate change causality.

No.	Q-sort statement	Factor array 1	Factor array 4
12	The climate is affected by the behaviour of people.	Agree	Agree
13	Increasing population growth causes climate change.	Agree	Disagree
14	Climate change is caused by technology.	Neutral	Disagree
15	Climate change is related to the burning of fossil fuels and pollution.	Agree	Strongly agree

Note: For further contextualisation of Table 5, refer to Appendix 3.

The narrative description of each factor is a shortened version of the Q-sort statements that scored significantly in the factor arrays. Thus, the narrative serves as a description of the worldview or attitudes held by respondents as related to the research topics of beliefs and climate change. Additionally, each identified factor (worldview) is also subjectively labelled by the researchers to facilitate easier distinction between the five factors, for example, Factor 1 – Collectivist or Liberal; Factor 2 – Religious Determinist or Fatalist; Factor 3 – Religious; Factor 4 – Technology or Human; and Factor 5 – Governance or Structural.

Based on the analysis of the five factor arrays, Factor 1 – Collectivist or Liberal (hereafter referred to as F1) and Factor 4 – Technology or Human (F4) are significantly representative of the Q-sort statements relevant to *Afro-polychronism* (indigenous collectivistic SSC and TO). Furthermore, F4 predominantly represents the Q-sort statements linked to climate change awareness and causality. As such, it is F1 and F4 that emerged as the most relevant factors (worldviews) when investigating *Afro-polychronism* and how it influences climate change adaptation in the South African context. The narrative description of F1 and F4 are as follows:

A qualitative approach is utilised to interpret why the statements may have scored accordingly in these factor arrays. By doing this, the researchers aim to better explain what cultural beliefs (TO and SSC) are held by respondents and to facilitate better cognisance as to how these beliefs influence human behaviours (more specifically, climate change adaptation). The quality of the interpretation step is based on the skills of the researchers who make deductions from ‘accumulated knowledge, expertise and intuition to interpret the viewpoints that emerge through factor analysis, closely guided by the particular configuration of items in each typal [*sic*] array…’ (Davis & Michelle 2011:571).

A qualitative analysis of the 12 Q-sort statements relevant to *Afro-polychronism* (SSC and TO), and climate change awareness and causality, will be based on how these statements scored in factor arrays 1 and 4. This analysis will be supported by the conceptual framework presented in this article.

### Q-sort statements related to Afro-polychronic collectivism

As a point of departure to investigate *Afro-polychronism* and how it influences behaviour such as adaptation to climate change, it is cardinal to establish if *Afro-polychronism* (more specific the SSC collectivistic trait) is in fact represented by the respondents’ worldviews. This is done through the qualitative analysis of Q#27, Q#35 and Q#22 based on the statements’ score in factor arrays 1 and 4.

As mentioned, the SSC collectivistic trait of relational rationality is a dominant value of *Ubuntu* as found in *Afro-polychronism*. Respondents are in agreement with Q#27, ‘*We can solve climate problems when we stand together and unite*’ and Q#35 ‘*In order to change our beliefs about the climate, we must sit down and discuss the matter*’. As such, Q#27 and Q#35 indicate respondents’ agreement to the interaction and participation between individuals is inferred by the term ‘we’. This term can be interpreted diversely by subjective identification regarding *which* individuals will make up the ‘we’ group. The respondents might have understood the term ‘we’ as representative of their community specifically, rather than humanity as a collective. This may be because of the cultural Afro-polychronic SSC trait of collectivism found in *Ubuntu* where the focus is allocated to identity as ‘I’ in ‘We’. Members identify themselves as part of the communal ‘We’, which in turn motivates behavioural coordination to attain shared goals, for example, finding solutions to climate problems and to change beliefs about climate. Supportively, a respondent stated that:

‘… culture is a strong part of the people’s lives because it influences people’s views and values, as well as their loyalties, so talking of culture…. We are talking about a group of people who are working together in reaching a common goal.’ (01-KM-Jouberton)

Shared goals can be achieved through solidarity, which is focal in *Ubuntu* collectivism. Here members of a group will provide mutual aid and positive attitudes to reach goals by working together.

Furthermore, respondents’ agreement (F4) with Q#22, *Young people can help older people catch up with new knowledge about the climate*, is indicative of the collective relationship orientation and group responsibility in *Afro-polychronism*. Interestingly, Q#22 did not rank significant in F1 like Q#27 and Q#35. Nonetheless, a neutral score does not automatically indicate a negative attitude towards Q#22. Instead, it points to the priority allocated to Q#22 during the Q-sorting forced-distribution process.

The neutral ranking of Q#22 in F1 might signal that the collectivistic trait represented by this statement was interpreted differently compared to Q#27 and Q#35. Here, Q#22 focuses on the relationships between generations. This may be explained by William Conton (1966) when he asserts that:

Africans generally have deep and ingrained respect for old age, and even when we can find nothing to admire in an old man, we will not easily forget that his grey hairs have earned him right to courtesy and politeness. (p. 21)

Supportively, a respondent stated that:

‘… it’s going to be difficult [for young people to educate older people about climate change], because, according to me, in order for old people to listen to you, you have to convince them, you have to prove that this is like this.’ (05-RM-Jouberton)

Hence, the neutral ranking of Q#22 in F1 may be because of the cultural respect allocated to elders.

Abductively, Q#27, Q#35 and Q#22 are representative of attitudes linked to the Afro-polychronic SSC trait of collectivism *as per* the focus on quality interpersonal relationships and the shared responsibility allocated to group members regarding climate change adaptation.

### Q-sort statements related to Afro-polychronic time orientation

Three Q-sort statements (Q#18, Q#19 and Q#1) pertain to Afro-polychronic TO and the qualitative interpretation of these Q-sort statements’ factor arrays is expressive of the TO held by the respondents.

Respondents reacted negatively to Q#18, *The climate was not better when I was younger* (F1 and F4). Ergo, according to the respondents’ interpretation, the climate was, in fact, better in the past when compared with the present. When referring to why the climate is perceived as being better in the past, a respondent stated that:

‘I think is was better. I mean, I remember when we were younger we could predict when it was gonna [sic] rain and all of that stuff, you know. Now you never know. Even things are worse now.’ (AB-01-Ikageng-P3)

This TO is focused on the past which coincides with the Afro-polychronic notion that time is constituted by present and past experiences and that time flows in a backward direction. This is on par with *Afro-polychronism*, where the past and present are a unified timeframe.

Representative of respondents’ like-mindedness is the positive attitude to Q#19, *We can solve environmental problems by returning to the ways of the past* (F4). By agreeing that solutions for environmental issues can be addressed by referring to past behaviour, Q#19 represents *Afro-polychronism* whereby wisdom that can be used in the present is derived from past experiences. The future is perceived in a near-sighted manner as it has not occurred yet and can thus provide no wisdom that can be utilised in the present. Additionally, a sense of belonging (being-with-others) is created when past experiences are shared by individuals in the present, and this Afro-polychronic TO contributes to a collective SSC (as prevalent in *Ubuntu*).

Based on F1, respondents reacted neutrally to Q#1, *The climate is a natural part of the world we just have to accept and live with. Afro-polychronism* has a near-sighted future view where the future is uncontrollable when perceived in a far-sighted manner.^3^ When discussing climate as an unavoidable and natural occurrence to be accepted, a respondent supportively stated:

‘I won’t say that this year it will snow because – like last year it happened whereby we didn’t expect it, but just because it happened, you must deal with it. Cause even now, if again – we’re not sure of that. Like I said, we can’t change nature.’ (05-AB-Jouberton)

Derived from the researchers’ interpretation, it is abductively argued that Q#18, Q#19 and Q#1 are representative of Afro-polychronic TO whereby the present and past constitute a unified timeframe. The attitudes towards Q#18 and Q19 may also support the Heideggerian supposition of *Vergangenheit Vergesenheit* (the past, forgotten), where the past and present are inseparable (unified timeframe in *Afro-polychronism*) to such a degree that the past should be remembered to suppress the degradation of the present. Supportively, shared experiences in this unified timeframe provide wisdom to be utilised regarding solutions to climate change issues.

### Q-sort statements related to climate change awareness

When investigating whether *Afro-polychronism* might influence climate change adaptation, it is crucial to establish if the respondents are indeed aware of climate change. Awareness can influence the risk perception of the group, which in turn may motivate adaptive behavioural change. Perception of risk will also be influenced by cultural beliefs such as TO and SSC. Q#4 and Q#5 are representative of climate change awareness.

Interestingly, respondents reacted negatively to Q#4, *The climate is not changing* (F1, and F4). Ergo, the respondents feel that the climate is changing. Cognisance of the change in climate is illustrated by statements such as: ‘But this time, it is possible to find the temperature to be still high in winter. Hot. The current heat and that of the past are different’ (06-RM-Jouberton), and *‘… sometimes winter, you know, it starts late there June, but sometimes it starts earlier than that. That … I believe that I can see the climate change*’ (01-AB-Jouberton).

Q#5, *There’s something wrong with the climate*, was met with agreement by respondents (F4). When considering that the North-West Province in South Africa (where the research was conducted) receives summer rainfall predominantly from October to April followed by cold and dry winters, respondents indicated abnormality in experienced climate by asserting:

‘Okay, according to my view climate is no like it used to be, nowadays during winter there can be rain’ (05-KM-Ikageng)

and

‘Because now we see rains are a bit more hectic than we’re used to. They’re coming at times when we’re not used to them coming. It’s getting a bit colder than I’m used to.’ (04-SS-Ikageng)

The respondents’ opinions indicate that they are currently aware of climate change based on present and past experiences of climatic change. Additionally, referring to the past to evaluate climatic experiences of the present is indicative of the unified present–past timeframe of *Afro-polychronism*. As such, the past is not forgotten, and erstwhile experiences of climate serve as a source of wisdom when assessing the present. Conclusively, *Afro-polychronism* does have an influence on how the respondents’ evaluate and show awareness of climatic changes which in turn will affect risk perception.

### Q-sort statements related to climate change causality

In conjunction with the Q-sort statements relating to climate change awareness, four Q-sort statements (Q#12, Q#13, Q#14 and Q#15) were identified to measure the respondents’ opinions regarding causes of climate change.

Q#12, *The climate is affected by the behaviour of people*, focuses on possible anthropogenic actions as contributing to climatic change, with which respondents’ agreed (F1 and F4). Q#13, *Increasing population growth causes climate change*, implies that an increase in human population will lead to more human behaviour contributing to climatic change. Interestingly, respondents disagreed with this statement (F4). Based on the aforementioned attitudes towards Q#12 and Q#13, the contradiction is clear: Increasing population growth (which will lead to more anthropogenic behaviour) does not cause climate change regardless of agreement that the climate is affected by anthropogenic behaviour (F4).

Abductively, the nonspecific nature of the term *people* in Q#12 might have been interpreted as referring to no particular group of humans. Based on Afro-polychronic collectivism (SSC), the respondents may not identify with the behaviour of people who are not considered part of their communal ‘We’. This may enable the respondents to dissociate themselves from contributors to climatic change.

The focus of Q#13 is on population growth as a contributor to climatic change and not on the specific actions of groups, which may explain the respondents’ disagreement (F4). The respondents might have been unable to identify with the term *population* based on the Afro-polychronic collectivistic group orientation whereby members of a group identify as ‘We’. *Population* might have been interpreted as consisting of various groups, thus enabling the respondents to distance their group from the concept. It might also be that the respondents deem population growth (maybe interpreted as an increase in members of a specific group) as positive as a result of *Afro-polychronism*’s emphasis on quality and timely interpersonal relationships of the ‘We’ group.

The above contradiction might be ascribed to the open-endedness of the term *behaviour* (Q#12). Without reference to specific anthropogenic behaviour, the respondents might have been less inclined to identify the relation between human behaviour, population growth and climate change. Additionally, behavioural impacts in the ‘far’ future may be less obvious in reference to the Afro-polychronic near-sighted future orientation where limited credit and emphasis are allocated to future occurrences (TO). As such, there might be a lack of cognisance of or attention to population growth and the subsequent increase of anthropogenic behaviour in the future, which might have an impact on climate change that will be experienced in a distant future timeframe. Abductively, the relation between population growth and increased human behaviour that contributes to climate change may be unclear to respondents, which might explain the preceding contradiction (F4).

Q#14, *Climate change is caused by technology*, states that technology (i.e. scientific advances, modern inventions and optimal industrialised practices) contributes to climate change. Respondents were neutral towards (F1) and in disagreement (F4) with this statement. This might be explained by the reflections some respondents made regarding technology: ‘This is modern technology, cell phones and other things’ (RM-02-Jouberton-P2) and ‘No. How by technology? Because technology is things like phones, and they are not related to the climate’ (IM-02-Jouberton-P2).

Interpretively, technology does not represent scientific advances and efficient modern industrial processes. Instead, it is regarded as concrete and tangible daily objects, for example, cellular phones. The objectification of technology may clarify why the respondents are of the opinion that the use of technology does not affect climate change. The concept technology may also have been interpreted as progress and advancement into the future. *Afro-polychronism* (TO) dictates that future events cannot constitute time, and therefore, this notion of technology (as representative of progression) stands in contrast with the unified past–present timeframe. Progress (created by technology) symbolises the changeability of future events and the objectification of technology places it in the present as a tangible item and thus dissociates it from the Afro-polychronic temporal view.

Respondents were in agreement with Q#15, *Climate change is related to the burning of fossil fuels and pollution* (F1 and F4). Here the use of fossil fuels and subsequent pollution are laid at the door of industrial activities, thus excluding specific groups’ behaviour as contributors. Supportively, a respondent stated:

‘In many factories, they are manufacturing maybe paper… plastic like this one, … motor parts… So it has anything or everything to do with pollution, because where they are producing; there’s going to be smoke that comes and pollute the air.’ (03-AB-Jouberton)

By implying that factories burn fossil fuels and consequently create greenhouse gasses that lead to increased pollution which affect the climate, the respondents are able to distance themselves and their group (‘We’) from having prominent responsibility. This distancing may be motivated by Afro-polychronic collectivism (SSC).

During the semi-structured interviews, automobiles are also mentioned as burners of fossil fuels as supported by the assertion that ‘there are lot of gases coming from a lot of cars’ (02-SS-Ventersdorp). Utilising public transport is common practice in peri-urban areas as a result of the low socio-economic status, which makes it challenging to own private transport. This may help the respondents to dissociate as contributors to climate change because they are not driving the public transport vehicles. The pollutants can, therefore, be regarded as ‘outsiders’ who are not included in the respondents’ group (‘We’) by differentiating public transport passengers from the people accountable for driving or owning public transport vehicles.

Conclusively, the aforementioned abductions signal that *Afro-polychronism* (TO and SSC) has an influence on the perceived causality of climate change.

### Overall correlation between statements in factors 1 and 4

F1 and F4 weightily constitute 30% of the sample variance. Based on the analysis of factor arrays, F1 and F4 are significantly representative of the Q-sort statement relevant to *Afro-polychronism* (SSC and TO). Supportively, Q-sort statements pertaining to *Afro-polychronism*, climate change awareness and causality (with the exception being #1 *The climate is a natural part of the world we just have to accept and live with*) indicated a high-scoring factor array in either F1 or F4; with Q-sort statement #27, *We can solve climate problems when we stand together and unite*, scoring high in both factors.

In F1 and F4, there are statements where the respondents reflected similar attitudes (either negatively or positively), with these being Q#27, *We can solve climate problems when we stand together and unite* (positive); Q#35, *In order to change our beliefs about the climate, we must sit down and discuss the matter* (positive)*; Q*#18, *The climate was not better when I was younger* (negative); Q#4, *The climate is not changing* (negative); Q#12, *The climate is affected by the behaviour of people* (positive); and Q#15, *Climate change is related to the burning of fossil fuels and pollution* (positive).

### *Afro-polychronism*: Influences on climate change adaption

*Afro-polychronism*, as represented by the attitudes held by respondents in peri-urban South African communities, is supported by the results of the factor array analysis, the qualitative interpretations of Q-sort statements, the statements made by respondents and the abductive reasoning presented. *Afro-polychronism* encapsulates the unique cultural beliefs of TO and SSC and this worldview influences the risk perception of the respondents and the consequential motivation for behavioural change.

*Afro-polychronism* focuses on a unified past–present orientation, with a near-sighted future view. This temporal view is supported by the respondents’ opinion that the climate was better in the past and that solutions for climate change can be found by returning to the ways (behaviour) of the past. This might provide a challenge when motivating the community to act in the present by evaluating the consequences current actions will have on future climatic changes.

It is clear that the respondents are aware of climate change. Nonetheless, it is possible that the respondents dissociate themselves as contributors to climate change, based on some attitudes and beliefs regarding the causality of climate change. This dissociation may be attributed to the collectivistic nature of *Afro-polychronism* where members of a group identify as a specific ‘We’ and those perceived as predominantly responsible for contributing to climate change are considered ‘outsiders’. Thus, climate change awareness may not provide enough motivation for communities to adapt their behaviour. It is therefore imperative to investigate and include a community’s worldview when implementing climate change adaptation strategies as the culture of a community will dictate the behaviour of said community.

Additionally, Afro-polychronic collectivism has the potential to be utilised in a positive manner when motivating behavioural changes. Here identity and solidarity are important traits of collectivism whereby the members of a community who identify as ‘We’ will be motivated to coordinate behaviour to attain shared goals, for example, raising climate change awareness to motivate the adoption of adaptive strategies. Such shared goals can be attained through solidarity where ‘We’ exhibit mutual support.

Strong Afro-polychronic collectivism also provides another opportunity to facilitate the adequate risk perception needed to motivate behavioural change. The collective ‘We’ is based on relationality and even though *Afro-polychronism* is predominantly past–present orientated with a limited future view, traits of identity and solidarity can be used. To highlight possible risks faced by the community, awareness regarding the effects of climate change on the children and future generations of the community should be communicated. This has the potential to motivate collective action for the betterment of the group.

### Suggestions based on the identified need for climate change awareness

A community’s culture will determine the beliefs of the group as illustrated by the respondent’s statement: ‘You have a culture. And it ends there. You will have to practice your culture. That is what will make you and that’s what is you’ (04-AB-Jouberton). These beliefs will influence the community’s risk perception when faced with climate change; hence, the investigation of cultural beliefs is of importance as a level of risk must be perceived to identify risks before context-specific adaptation strategies can be developed. However, to identify risks, DRR information must be present as the ‘socially influenced risk perception will be subjected to the rational ascribed to preceding hazardous events in order to support the cultural group’s harmony’ (Van Niekerk & Terblanche-Greeff 2017:11).

It is clear that some of the respondents are aware of climate change and its causes. During the semi-structured interviews, some of the respondents identified the need for information regarding DRR to motivate adaptive behavioural change. The lack of cognisance of the consequences of anthropogenic behaviour pertaining to climate change is illustrated by a respondent’s assertion:

‘What I could say – many people they don’t understand, once you’ve thrown the paper around the field … that causes the climate. Once, maybe you’re burning the fire around the environment you are doing the climate. People are not aware of that. But once, maybe you could take the people and sit down and motivate them to try to learn more about it, maybe it could solve the problem.’ (04-SS-Jouberton)

This quote illustrates that DRR information to raise awareness regarding climate change is identified as a possible solution to motivate change in the community’s behaviour. Based on the recognised need and the fact that a community’s culture will greatly influence risk perception and behavioural change, it is proposed that the community-based disaster risk management approach should be implemented. The participatory nature of this approach will enable the community to become part of the knowledge-sharing process. This approach also promotes the inclusion of the community with regard to the gathering of information pertaining to the specific culture of the community and the climate change risks faced by community members.

By including the community, the DRR information provided to facilitate risk perception – which consequently can motivate behavioural change – should be based on the cultural beliefs of the community. The tailoring of DRR information may enable the community to more easily identify with the information which will motivate them to take ownership of the DRR process.

## Conclusion

The focus on cultural beliefs is crucial when investigating a community’s adaptation to climate change. This article presents results that indicate how cultural Afro-polychronic SSC and TO influence attitudes towards climate change awareness and causality. Subsequently, these beliefs also influence the community’s risk perception and priority allocated to behavioural changes needed for adaptation.

The effects of climate change present over extended periods of time and this necessitates current behaviour to mitigate and adapt thereto. However, the perceived need for action in the present will be influenced by a community’s future orientation as possible future occurrences must be imagined or anticipated to motivate current behavioural changes.

In *Afro-polychronism*, the past and present are seen as a unified timeframe, paired with a limited future orientation. Climate change causality and awareness are evaluated and based on this unified timeframe. As adaptation requires the acknowledgement of possible future events caused by anthropogenic behaviour, this near-sighted future orientation limits cognisance thereof. Additionally, the future orientation needed for climate change adaptation might not be prevalent in *Afro-polychronism* as the future has not been experienced, and thus, cognitive projection into this timeframe is of little value. This lack of focus on possible future situations hampers the community’s risk perception, which can have dire effects on climate change adaptation.

Furthermore, based on the SSC beliefs held by the community, the respondents identify themselves as ‘I’ in ‘We’, which is prevalent in Afro-polychronic collectivism. This enables them to dissociate themselves (communal; ‘We’) as contributors to climate changes, which also limits risk perception when responsibility for anthropogenic behaviour that contributes to climatic changes is allocated to ‘others’.

Interestingly, the respondents are aware of climate change and identified the need for more information regarding DRR. However, mere awareness of climate change is not sufficient to motivate behavioural changes. Instead, a community must make sense of the provided information from the vantage point of their cultural beliefs. The logic allocated to the information will influence the risk perception of the community. Deductively, it is cardinal to develop DRR information and adaptation strategies based on cultural beliefs such as *Afro-polychronism*.

## Recommendations for future studies

Q-methodology does not provide results that can be used as a generalisation and, when considering that culture is context-specific, it is recommended that more Q-studies should be conducted on the multitudinous cultural groups in South Africa to measure beliefs regarding climate change. These cross-cultural studies can be used to investigate and compare how SSC and TO influence climate change adaptation. Subsequently, unique DRR information and adaptive strategies can be tailored for specific cultural communities to motivate ownership of needed behavioural change.

## Figures and Tables

**FIGURE 1-A3:**
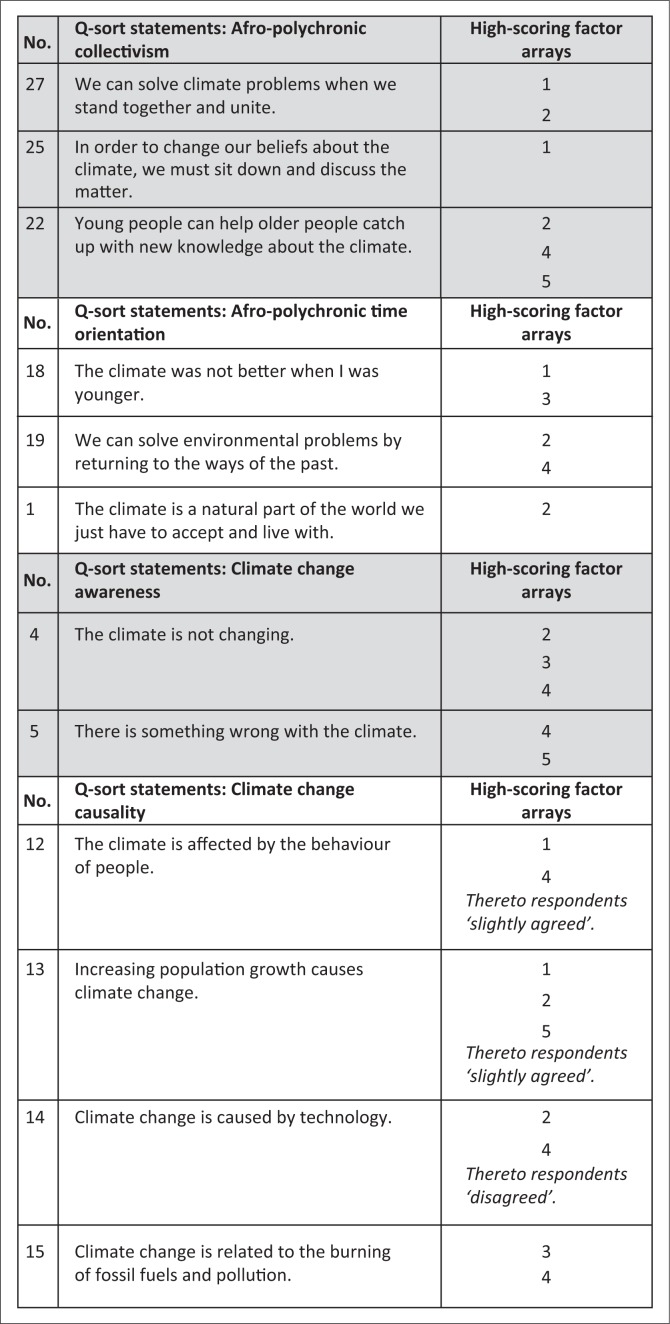
Q-sort statements pertaining to *Afro-polychronism* and climate change.

## Footnotes

1 P-sample (i.e. person-sample) is the group of respondents selected to perform the task of Q-sorting.

2 See Appendix 1 for the complete Q-sample.

3 The neutral score indicates the priority allocated to the statement during the forced-distribution Q-sort process.

## Appendix 1: Q-sample

1. The climate is a natural part of the world we just have to accept and live with.
2. The climate is complicated.
3. The climate is unpredictable.
4. The climate is not changing.
5. There is something wrong with the climate.
6. Climate change is not a sign that the world is ending.
7. Natural disasters happen when nature wants to reshape itself.
8. The climate is determined by God.
9. Climate change is not punishment for the sins that people commit.
10. Climate change is caused by the fighting of the ancestors.
11. Traditional healers cause the climate to change.
12. The climate is affected by the behaviour of people.
13. Increasing population growth causes climate change.
14. Climate change is not caused by technology.
15. Climate change is related to the burning of fossil fuels and pollution.
16. The climate influences the growth of crops and the production of food.
17. People are trying to make money, that’s why they are damaging the environment.
18. The climate was not better when I was younger.
19. We can solve environmental problems by returning to the ways of the past.
20. The next generation will be influenced by our current behaviour towards nature.
21. We must act now to prevent the climate problems of the future.
22. Young people can help older people catch up with new knowledge about the climate.
23. We have the right to know about climate issues that affects us directly and indirectly.
24. Educating people about climate change will anger the ancestors and cause bad luck.
25. It is not the duty of the government to inform people about climate change.
26. We can address climate problems by drafting laws that protect the environment.
27. We can solve climate problems when we stand together and unite.
28. It is possible for humans to control the climate through technology.
29. Using sustainable technology is not good for the climate.
30. It is difficult to care about climate change because of economic pressures.
31. The climate does not play an important role in our lives.
32. We do not have to respect the environment.
33. It is difficult to educate people about climate change because of their beliefs.
34. It is possible to change my beliefs when someone else tells me to.
35. In order to change our beliefs about the climate, we must sit down and discuss the matter.
36. My beliefs can change if I see in reality that things are different from my beliefs.
37. My beliefs about the climate can change when mechanisms are in place to protect us.
38. I am open to change my beliefs, because I learn new things all the time.
39. It is not possible to change my beliefs.
40. The climate influences how people feel emotionally and that may cause changes in their beliefs.

## Appendix 2: Description of factors

**TABLE 1-A2:** Factors.

Factor (worldview)	Description or factor narrative
Factor 1: Collectivist or liberal	‘The climate change we experience today is not a punishment for people’s sins and neither is it a sign that the world is ending, but rather a natural occurrence where nature wants to reshape itself. Since the climate affects how people’s emotions, it can also cause people to change their beliefs. If we unite we have better chance at solving climate problems and influencing the next generation’s attitudes towards nature. This is important since the climate influences the growth of crops and production of food, and we have to act now to prevent further changes to the climate’.
Factor 2: Religious determinist or fatalist	‘The climate can’t be changed by traditional healers since it’s determined by God. Because of this, the climate is just a natural part of the world that we have to accept and it is not affected by people’s behaviour. Climate change is not related to the burning of fossil fuels, climate is just unpredictable. I’m open to changing my beliefs, but I think the best way of solving any possible environmental problems is by returning to the ways of our ancestors. Things were better when I was younger, and I think that today’s technology plays an important part in the changes we see. In others words, I don’t believe that there is anything wrong with the climate and I don’t think environmental problems are a sign that the world is ending’.
Factor 3: Religious	‘The climate is determined by God and climate change is a sign that the world is ending. I will not change my belief and the climate influences neither me nor other people emotionally. The climate is not that complicated seeing as the changes are mainly related to the burning of fossil fuels. This means that we can also control the climate through technology, and that natural disasters are largely caused by people’s actions. The next generation will be influenced by our behaviour towards nature, but that doesn’t mean we should return to the ways of old. The climate was not better when I was younger, and I think young people can help the older generations get educated about climate change. This is important, since we have to act now to hinder further damage to the environment’.
Factor 4: Technology or human	‘The climate plays an important part in our lives and we need to respect the environment. We have the right to know about climate issues that affect us directly and indirectly. Even though the climate is changing, it’s not caused by population growth and is not a sign that the world is ending. Sometimes I think that traditional healers can cause the climate to change, but I also believe that the changes in the climate are related to the burning of fossil fuels and people damaging the environment when they are trying to make money. This means that we should rather try to use sustainable technologies, since this would benefit the environment. It may not be possible for humans to control the climate through technology, but if we work together, we can make a difference’.
Factor 5: Governance or structural	‘There is something wrong with the environment, but returning to our old ways is not the way to solve problems. Seeing how the climate is both complicated and unpredictable, the government plays an important part in informing people about the changing and drafting laws as an effective way of protecting the environment. There is no way around the fact that the changes we see today are consequences of people’s behaviour, and if we continue the way we are now we will destroy the earth. However, it might be difficult to educate people about the problems because of their beliefs, but I for one am open to changing what I believe to be true’.
